# Non-Enhanced MR Imaging of Cerebral Aneurysms: 7 Tesla versus 1.5 Tesla

**DOI:** 10.1371/journal.pone.0084562

**Published:** 2014-01-06

**Authors:** Karsten H. Wrede, Philipp Dammann, Christoph Mönninghoff, Sören Johst, Stefan Maderwald, I. Erol Sandalcioglu, Oliver Müller, Neriman Özkan, Mark E. Ladd, Michael Forsting, Marc U. Schlamann, Ulrich Sure, Lale Umutlu

**Affiliations:** 1 Erwin L. Hahn Institute for Magnetic Resonance Imaging, University Duisburg-Essen, Essen, Germany; 2 Department of Neurosurgery, University Hospital Essen, Essen, Germany; 3 Department of Diagnostic and Interventional Radiology and Neuroradiology, University Hospital Essen, Essen, Germany; Northwestern University Feinberg School of Medicine, United States of America

## Abstract

**Purpose:**

To prospectively evaluate 7 Tesla time-of-flight (TOF) magnetic resonance angiography (MRA) in comparison to 1.5 Tesla TOF MRA and 7 Tesla non-contrast enhanced magnetization-prepared rapid acquisition gradient-echo (MPRAGE) for delineation of unruptured intracranial aneurysms (UIA).

**Material and Methods:**

Sixteen neurosurgical patients (male n = 5, female n = 11) with single or multiple UIA were enrolled in this trial. All patients were accordingly examined at 7 Tesla and 1.5 Tesla MRI utilizing dedicated head coils. The following sequences were obtained: 7 Tesla TOF MRA, 1.5 Tesla TOF MRA and 7 Tesla non-contrast enhanced MPRAGE. Image analysis was performed by two radiologists with regard to delineation of aneurysm features (dome, neck, parent vessel), presence of artifacts, vessel-tissue-contrast and overall image quality. Interobserver accordance and intermethod comparisons were calculated by kappa coefficient and Lin's concordance correlation coefficient.

**Results:**

A total of 20 intracranial aneurysms were detected in 16 patients, with two patients showing multiple aneurysms (n = 2, n = 4). Out of 20 intracranial aneurysms, 14 aneurysms were located in the anterior circulation and 6 aneurysms in the posterior circulation. 7 Tesla MPRAGE imaging was superior over 1.5 and 7 Tesla TOF MRA in the assessment of all considered aneurysm and image quality features (e.g. image quality: mean MPRAGE7T: 5.0; mean TOF7T: 4.3; mean TOF1.5T: 4.3). Ratings for 7 Tesla TOF MRA were equal or higher over 1.5 Tesla TOF MRA for all assessed features except for artifact delineation (mean TOF7T: 4.3; mean TOF1.5T 4.4). Interobserver accordance was good to excellent for most ratings.

**Conclusion:**

7 Tesla MPRAGE imaging demonstrated its superiority in the detection and assessment of UIA as well as overall imaging features, offering excellent interobserver accordance and highest scores for all ratings. Hence, it may bear the potential to serve as a high-quality diagnostic tool for pretherapeutic assessment and follow-up of untreated UIA.

## Introduction

Rupture of intracranial aneurysm is associated with high morbidity and mortality rates, as it is known to be accountable for 80% of all subarachnaoid hemorrhages (SAH), causing 25% of all cerebrovascular-related deaths, [1]. Size and shape of unruptured intracranial aneurysms (UIA) are known to be significantly affiliated with rupture rates, hence, high-quality assessment of UIA and its related features displays an important role on potential aneurysm treatment [2]–[4].

Digital subtraction angiography (DSA) is considered the gold standard for detection of UIA. Nevertheless, due to the application of ionizing radiation and iodinated contrast agent as well as the general risk affiliated to invasive interventional procedures, DSA is associated with a 0.2%–0.5% risk for severe permanent neurological complications [5], [6].

Within the past 15 years, 1.5 Tesla magnetic resonance angiography (MRA) has evolved to become an excellent non-invasive diagnostic alternative to DSA, yielding sensitivity rates of 79–97% for the detection of small UIA [7]–[10]. With the successful introduction of (ultra-) highfield non-enhanced MRA of the intracranial vasculature, recent studies performed at 3 and 7 Tesla reported improved depiction of UIA with sensitivity rates comparable to the gold standard DSA [11]–[14]. The increase of the magnetic field strength from 1.5 to 3 Tesla, and respectively to 7 Tesla, allowed for a successful transition of the increased signal-to-noise (SNR) and contrast-to-noise ratio (CNR) to improvements in spatial resolution and vessel contrast.

The purpose of this prospective study was to evaluate the image quality and diagnostic ability in the assessment of UIA of 1.5 Tesla TOF MRA in comparison to ultra-high-field TOF MRA and non-enhanced MPRAGE imaging.

## Materials and Methods

### Ethics Statement

The study was conducted according to the principles expressed in the Declaration of Helsinki and was approved by the authorized ethical review board of the University Duisburg-Essen. Written informed consent was obtained before each examination.

### Study Design and Population

This prospective study evaluates the diagnostic ability of 7 Tesla TOF MRA in comparison to 1.5 Tesla TOF MRA and 7 Tesla non-contrast enhanced MPRAGE for delineation of UIA. The study group comprised 16 neurosurgical patients (male n = 5, female n = 11, average age 53.38 years; range 38–70 years). Inclusion criteria were: 1) single or multiple UIA, 2) age 18–80 years, 3) ability to give informed consent and 4) legal competence. Exclusion criteria were: 1) cardiac pacemakers or any other electronic implants, 2) metallic implants, 3) pregnancy or breast feeding period, 4) claustrophobia and 5) chronic or episodic vertigo. All patients were accordingly examined at a 7 Tesla (Magnetom 7T, Siemens) and a 1.5 Tesla MR scanner system (Espree, Siemens) utilizing dedicated head coils. The following sequences were obtained: (1) 7 Tesla TOF MRA, (2) 1.5 Tesla TOF MRA and (3) 7 Tesla non-contrast enhanced MPRAGE. A total of 20 intracranial aneurysms were detected, with two patients showing multiple aneurysms (2, respectively 4 aneurysms).

### Scanners and Coil Systems

Ultra-high-field examinations were acquired on a 7 Tesla whole-body MRI system (Magnetom 7T, Siemens Healthcare, Erlangen, Germany) utilizing a 32-channel Tx/Rx head coil (Nova Medical, Wilmington, USA). The scanner is equipped with a gradient system of 45 mT/m maximum amplitude and a slew rate of 200 mT/m/ms.

Concomitant 1.5 Tesla examinations were acquired on a whole-body MRI system (Espree, Siemens Healthcare) equipped with a 12-channel Rx head coil (Siemens Healthcare, Erlangen, Germany). The scanner is equipped with a gradient system of 33 mT/m maximum amplitude and a slew rate of 200 mT/m/ms.

### Examination at 7 Tesla

Prior to the acquisition of the diagnostic sequences B_0_ shimming was performed using a vendor-provided gradient echo sequence and algorithm based on the work of Schar [15]. For B_1_ field mapping and local flip angle optimization a vender provided spin-echo type sequence was used. After a slice selective excitation, two refocusing pulses generate a spin-echo and a stimulated echo, respectively. The algorithm is mainly based on the work of Hoult [16].

### TOF MRA sequence at 7 Tesla

The TOF MRA sequence is based on a 3D FLASH sequence with flow compensation and tilt-optimized non-saturated excitation (TONE) across the slab [17]. Datasets were acquired with an excitation flip angle of α = 18°, TE  = 4.34 ms, TR = 20 ms, FOV 200 mm ×169 mm ×46 mm, 112 slices per slab (oversampling 14%), GRAPPA acceleration factor R = 4 (phase direction), partial Fourier 6/8 in both slice and phase directions, matrix of 896×756 (non-interpolated), and a voxel size of 0.22×0.22×0.41 mm^3^ in a total acquisition time of 6 min 22 s. The variable-rate selective excitation (VERSE) algorithm {Conolly, 1988#1168} was used to reduce SAR contribution of excitation and venous saturation RF pulses {Schmitter, 2011#1484}. The flip angle of the saturation RF pulses was additionally reduced (α_SAT_  = 35° instead of 90° which is normally used) to further ameliorate SAR constraints [17].

### MPRAGE sequence at 7 Tesla

MPRAGE imaging was obtained with the following sequence parameters: TR = 2500 ms, TE = 1.54 ms, TI = 1100 ms, TA = 6 min 13 s, GRAPPA acceleration factor R = 2, excitation flip angle α = 7°, adiabatic WURST pulse for magnetization preparation [18], bandwidth  = 570 Hz/px, 256 slices per slab (slice oversampling 75%), matrix 384×336 (non-interpolated), FOV = 270×236 mm^2^, voxel size 0.7×0.7×0.7 mm^3^.

### TOF MRA sequence at 1.5 Tesla

The TOF MRA sequence was based on a clinically used standard 3D gradient echo sequence. Datasets were acquired with an excitation flip angle of α = 25°, TE = 7 ms, TR = 26 ms, matrix 512×448 (interpolated), FOV 180 mm ×157 mm, 3 slabs with 44 slices per slab (oversampling 18.2%) and a voxel size of 0.35×0.35×0.7 mm^3^ in a total acquisition time of 4 min 3 s.

### Image Evaluation

Image evaluation was performed separately and independently by two experienced radiologists on standard post-processing Picture Archiving and Communcation system (PACS) workstations (Centricity RIS 4.0i, GE Healthcare, USA). Both radiologists were blinded to image acquisition methods and intracranial pathologies. Visual evaluation was performed using 3D image reconstructions; all measurements were performed on 2D multi-planar reconstructions of the 3D datasets. The total number of aneurysms, the maximal diameter as well as the diameter of neck and dome of each aneurysm were assessed. For qualitative analysis the following features were evaluated, utilizing a five-point scale (5 =  excellent, 4 =  good, 3 =  moderate, 2 =  poor, and 1 =  non-diagnostic vessel delineation):

1. Delineation of aneurysm dome
2. Delineation of aneurysm neck
3. Delineation of parent vessel
4. Presence of artifacts
5. Vessel tissue contrast
6. Overall image quality.

Vessel-tissue contrast ratio 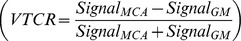
of the middle cerebral artery (MCA) was assessed in correlation to surrounding gray matter (GM) for 7 Tesla MPRAGE sequences, 7 Tesla TOF sequences and for 1.5 Tesla TOF sequences. Therefore, regions of interest (ROI) were placed in the largest diameter of the proximal left M1 segments 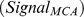 and adjacent gray matter 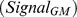. The average diameter for the ROI of the vessel was 3–5 mm; the ROI for brain parenchyma amounted to approximately 10 mm. 

Interobserver accordance for ordinal scale variables were rated using the kappa coefficient [19] (k) according to Landis [20] as follows: Poor (k<0.00), Slight (k = 0.00–0.20), Fair (k = 0.21–0.40), Moderate (k = 0.41–0.60), Substantial (k = 0.61–0.80), Almost Perfect (k = 0.81–1.00). Interobserver accordance for ratio scale variables were rated using Lin's [21], [22] concordance correlation coefficient. For intermethod comparison the Wilcoxon signed rank test was applied. Differences of continuous scaled variables were tested by Student's t-test.

Statistical analysis was carried out with the STATA software package (Stata/SE 12.1 for Mac (64-bit Intel), StataCorp, 4905 Lakeway Drive, College Station, Texas 77845 USA).

## Results

All 1.5 Tesla and 7 Tesla scans were performed successfully without any relevant side effects. Both readers identified twenty intracranial aneurysms in 1.5 Tesla and 7 Tesla TOF MRA and 7 Tesla MPRAGE imaging. Fourteen of the twenty intracranial aneurysms were located in the anterior circulation: middle cerebral artery (n = 7), anterior cerebral artery (n = 2), internal carotid artery (n = 4) and posterior communicating artery (n = 1). Six aneurysms were detected in the posterior circulation: basilar tip (n = 2), posterior cerebral artery (n = 2), posterior inferior cerebellar artery (n = 1) and superior cerebellar artery (n = 1). Two patients had multiple intracranial aneurysms (2, respectively 4 aneurysms). Ten of twenty identified aneurysms were defined as small (3–5 mm), five as medium-sized (6–10 mm), three as large (11–25 mm) and one was rated a giant cerebral aneurysm (>35 mm). The mean aneurysm size was 8.65 mm (Standard Error (SE) 1.823) for the 7 Tesla TOF MRA reading, 8.075 mm (SE 1.708) for the 1.5 Tesla TOF MRA reading and 8 mm (SE 1.826) for the 7 Tesla MPRAGE reading. Interobserver accordance measured by Lin's concordance correlation coefficient was substantial for 7 Tesla TOF MRA readings (ρc = 0.963) and 1.5 Tesla TOF MRA readings (ρc = 0.951) and moderate for 7 Tesla MPRAGE readings (pc = 0.922). Table 1 shows an overview on basic demographic data and aneurysm size and location.

**Table 1:** Basic demographic data and aneurysm size and location.

aneurysm	subject	sex	age	location	side	7 Tesla TOF	1.5 Tesla TOF	7 Tesla
						MRA	MRA	MPRAGE
						Ø in mm*	Ø in mm*	Ø in mm*
1	1	female	52	ICA	left	8	7.5	7
2	2	female	56	giant ICA	right	36.5	34	35.5
3	3	female	69	ICA	left	16.5	17	18
4	4	female	56	MCA	left	8.5	8.5	9
5				BT		2.5	2.5	2.5
6	5	male	70	BT		6.5	6.5	6.5
7	6	male	45	PCA	left	18	13.5	16.5
8	7	female	66	ACA	right	1.5	1	1.5
9				PICA	right	4	3	4
10				MCA	left	5	5	5
11				MCA	right	10.5	10	10.5
12	8	female	54	MCA	right	4.5	4.5	6
13	9	female	60	ICA	right	17	17	16.5
14	10	female	44	ACA	right	2.5	2.5	2.5
15	11	female	53	MCA	right	8	8	8
16	12	male	45	MCA	left	5	5	4.5
17	13	female	49	MCA	left	6	5.5	5
18	14	male	38	PCA	left	4.5	4.5	3.5
19	15	male	55	PcomA	right	4.5	3.5	2.5
20	16	female	42	SC	right	3.5	2.5	2.5

internal carotid artery (ICA).

middle cerebral artery (MCA).

basilar tip (BT).

posterior cerebral artery (PCA).

anterior cerebral artery (ACA).

posterior inferior cerebellar artery (PICA).

posterior communicating artery (PcomA).

superior cerebellar artery (SC).

mean diameter from both readers.

### Dome, neck and parent vessel

Mean score values of both readers' for delineation of the aneurysm dome were 4.5 (excellent) (SE 0.136) for 7 Tesla TOF MRA, 3.9 (good) (SE 0.109) for 1.5 Tesla TOF MRA and 4.8 (excellent) (SE 0.092) for 7 Tesla MPRAGE imaging. Wilcoxon matched-pairs two-sided signed-ranks test showed significant differences between 7 Tesla TOF MRA and 1.5 Tesla TOF MRA (p = 0.0042) ratings as well as between 7 Tesla MPRAGE and 1.5 Tesla TOF MRA (p = 0.0000) ratings. There were no significant differences between 7 Tesla TOF MRA and 7 Tesla MPRAGE (p = 0.1797) ratings.

The delineation of the aneurysm neck was rated 4.5 (excellent) (SE 0.128) in 7 Tesla TOF MRA, 3.8 (good) (SE 0.156) in 1.5 Tesla TOF MRA and 4.8 (excellent) (SE 0.156) in 7 Tesla MPRAGE MRI. Wilcoxon matched-pairs two-sided signed-ranks test showed significant differences between 7 Tesla TOF MRA and 1.5 Tesla TOF MRA (p = 0.0075) ratings, between 7 Tesla MPRAGE and 1.5 Tesla TOF MRA (p = 0.0005) ratings as well as between 7 Tesla TOF MRA and 7 Tesla MPRAGE (p = 0.0063) ratings.

Quantitative measurements for dome and neck diameter showed larger dome/neck ratio for 7 Tesla MPRAGE (p = 0.0597) and 7 Tesla TOF MRA (p = 0.0305) compared to 1.5 Tesla TOF MRA. There was no significant difference between dome/neck ratio in 7 Tesla MPRAGE and 7 Tesla TOF MRA (p = 0.5586).

In accordance with the delineation of the aneurysm dome and neck, 7 Tesla MPRAGE also offered best assessment of the parent vessel 4.8 (excellent) (SE 0.128). TOF MRA yielded good assessment of the parent vessel at both field strengths (1.5 Tesla TOF MRA mean: 4.4 (SE 0.129); 7 Tesla TOF MRA mean: 3.9 (SE 0.146).

Wilcoxon matched-pairs two-sided signed-ranks test showed significant differences between 7 Tesla TOF MRA and 1.5 Tesla TOF MRA (p = 0.0309) ratings, between 7 Tesla MPRAGE and 1.5 Tesla TOF MRA (p = 0.0075) ratings as well as between 7 Tesla TOF MRA and 7 Tesla MPRAGE (p = 0.0117) ratings.

Interobserver accordance was substantial to almost perfect for most readings, with slightly lower accordance (fair) for delineation of the dome for 1.5 Tesla TOF MRA and 7 Tesla TOF MRA. Details are shown in Table 2.

**Table 2:** Interobserver accordance (kappa-statistic).

	dome	neck	parent vessel	artifacts	vessel-tissue contrast	overall image quality
**7 T TOF MRA**	0.75	0.79	0.85	0.89	0.88	0.50
**1.5 T TOF MRA**	0.39	0.80	0.73	0.71	0.84	0.68
**7 T MPRAGE**	0.26	1.00	0.92	1.00	0.84	1.00

**According to Landis the kappa coefficient (k) was rated as follows:**

Poor (k<0.00), Slight (k = 0.00–0.20), Fair (k = 0.21–0.40), Moderate (k = 0.41–0.60).

Substantial (k = 0.61–0.80), Almost Perfect (k = 0.81–1.00).

Disagreements were weighted by 1– {(i–j)/(k–1)}2 where i and j index the rows and columns of the ratings by the two raters and k is the maximum number of possible ratings.

Table 3 shows mean ratings for delineation of dome, neck and parent vessel of both readers. Table 4 lists the combined readings of both raters for dome and neck diameter in mm and calculated dome/neck ratio for all aneurysms.

**Table 3:** Ratings for dome, neck and parent vessel delineation (mean ratings from both readers).

aneurysm	7 Tesla	1.5 Tesla	7 Tesla	7 Tesla	1.5 Tesla	7 Tesla	7 Tesla	1.5 Tesla	7 Tesla
	TOF MRA	TOF MRA	MPRAGE	TOF MRA	TOF MRA	MPRAGE	TOF MRA	TOF MRA	MPRAGE
	dome	dome	dome	neck	neck	neck	parent vessel	parent vessel	parent vessel
1	5	4	5	4	5	5	4	5	5
2	4.5	3	3.5	5	4	2	5	4	3.5
3	4	3	5	4.5	4	5	4	5	4
4	5	4	5	5	5	5	5	4.5	5
5	4.5	3.5	4.5	5	4	5	5	4.5	5
6	5	4	5	5	4	5	5	4	5
7	5	3.5	5	5	3	5	5	3	5
8	4	4	4.5	4.5	3.5	5	4	3	5
9	4	4.5	4	4	3.5	5	4	4	5
10	4.5	4.5	5	4.5	5	5	4.5	5	5
11	5	4	4.5	5	4.5	5	4.5	4	5
12	3	4	5	4	3	5	4	4	5
13	4	4	5	3	4	4	3	4	3
14	5	4	5	5	3.5	5	5	4	5
15	5	3.5	5	5	4	5	5	3.5	5
16	5	4.5	5	4	3	5	4	3	5
17	5	4.5	5	5	3	5	4	3	5
18	4	4	5	4	3	5	4	3.5	5
19	3.5	3	5	4	4	5	4	3.5	5
20	5	3.5	5	4	3	5	5	4	5
**mean**	**4.5**	**3.9**	**4.8**	**4.5**	**3.8**	**4.8**	**4.4**	**3.9**	**4.8**

5 =  excellent. 4 =  good. 3 =  moderate. 2 =  poor. and 1 =  non-diagnostic vessel delineation.

**Table 4:** Combined readings of both raters for dome and neck diameter in mm and dome/neck ratio (DNR) for all aneurysms.

Subject	7 Tesla	7 Tesla	7 Tesla	7 Tesla	7 Tesla	7 Tesla	1.5 Tesla	1.5 Tesla	1.5 Tesla
	MPRAGE	MPRAGE	MPRAGE	TOF MRA	TOF MRA	TOF MRA	TOF MRA	TOF MRA	TOF MRA
	dome	neck	DNR	dome	neck	DNR	dome	neck	DNR
1	6.5	3	2.167	7	3	2.333	6	3	2
2	35	11	3.182	34	11	3.091	34	10.5	3.238
3	10	4	2.5	10	3	3.333	9.5	3	3.167
4	8	2	4	8	3	2.667	8.5	3	2.833
5	2.5	2	1.25	2.5	2	1.25	2.5	2	1.25
6	6.5	2	3.25	6.5	2.5	2.6	6.5	2	3.25
7	15	2	7.5	15	2	7.5	13.5	2	6.75
8	1.5	1	1.5	1.5	1	1.5	1	1	1
9	4	2	2	4	2	2	3	2.5	1.2
10	5	2	2.5	5	2	2.5	5	2	2.5
11	10.5	3	3.5	10	3	3.333	10	3	3.333
12	4	2	2	4	1	4	4	2	2
13	16.5	4.5	3.667	17	5	3.4	17	5.5	3.091
14	2.5	2	1.25	2.5	2	1.25	2.5	2	1.25
15	8	2	4	8	2	4	8	2	4
16	4.5	2	2.25	4	1.5	2.667	4	2	2
17	4	2	2	4	2	2	4	2	2
18	3	1	3	3	1	3	3	2	1.5
19	2.5	1	2.5	3	1	3	3	1	3
20	2.5	2	1.25	3	2	1.5	2.5	2	1.25
**mean**			*2.763*			**2.846**			***2.531***

standard error  = 0.318, 95% confidence interval [2.098–3.428].

standard error  = 0.307, 95% confidence interval [2.203–3.489].

standard error  = 0.299, 95% confidence interval [1.906–3.156].

**Figure 1:**
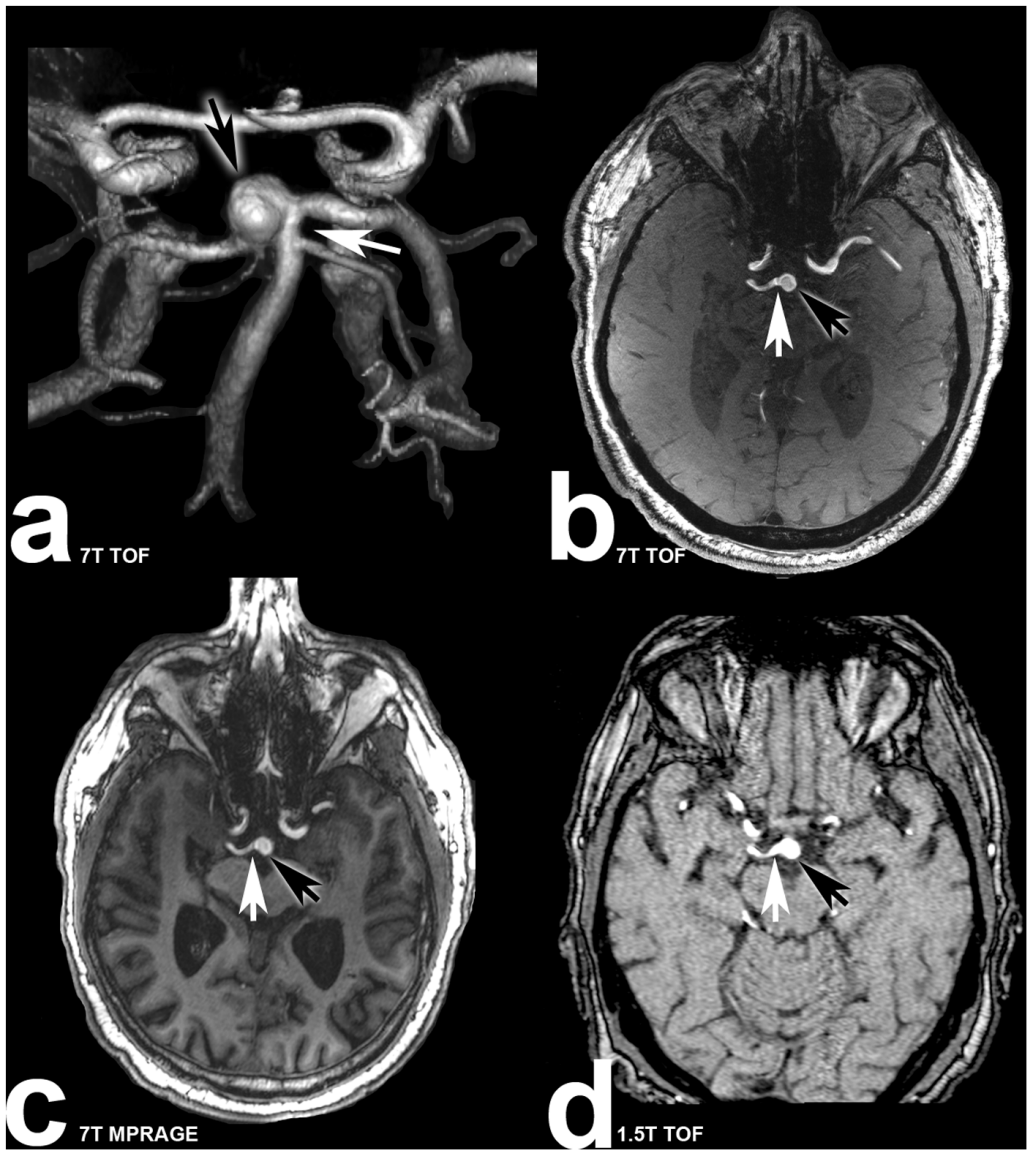
Seventy(subject 5) suffering from basilar tip aneurysm. White arrows are marking the aneurysm neck, black arrows are marking the aneurysm dome. **a:** Volume rendering of the 7 Tesla TOF MRA demonstrating the three dimensional structure of the aneurysm; **b:** transverse plane of the 7 Tesla TOF MRA clearly depicting the aneurysm dome, neck and parent vessel; **c:** 7 Tesla MPRAGE sequence with excellent delineation of the aneurysm; **d:** 1.5 Tesla TOF MRA scan with minor irregularities of the parent vessel and aneurysm dome.

**Figure 2:**
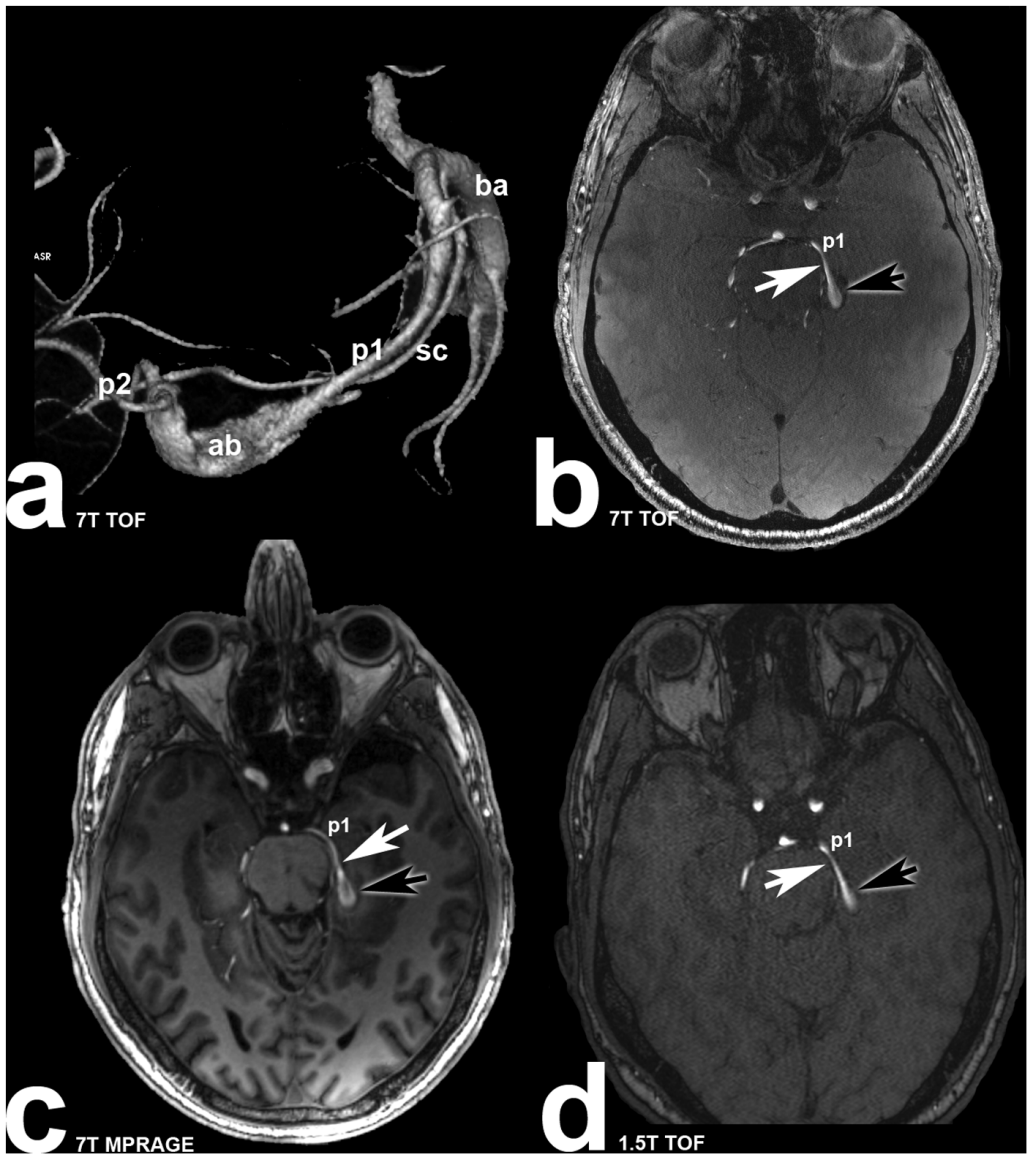
Forty-five years old male patient (subject 6) with partially thrombosed left sided fusiform posterior cerebral artery aneurysm. White arrows are marking the aneurysm neck, black arrows are marking the aneurysm dome. p1: posterior cerebral artery (p1 segment) **a:** Volume rendering of the 7 Tesla TOF MRA illustrating the three dimensional structure of the aneurysm (ba: basilar artery; sc: superior cerebellar artery; p2: posterior cerebral artery (p2 segment); ab: aneurysm body); **b:** transverse plane of the 7 Tesla TOF MRA depicting the parent vessel and part of the aneurysm body; **c:** Excellent delineation of parent vessel and part of the aneurysm body in 7 Tesla MPRAGE scan; **d:** Moderate depiction of parent vessel and aneurysm body in 1.5 Tesla TOF MRA.

Examples for aneurysm dome, neck and parent vessel delineation in all three MRI sequences are shown in Figures 1 and 2.

### Artifacts, vessel-tissue contrast and overall image quality

Seven Tesla MPRAGE imaging was the sequence to be least impaired by artifacts (excellent) with mean value of 4.9 (SE 0.000). 7 Tesla and 1.5 Tesla TOF MRA showed equivalent artifact impairment (good) with mean values of 4.3 (SE 0.147) for 7 Tesla and mean 4.4 (SE 0.124) for 1.5 Tesla imaging.

**Figure 3:**
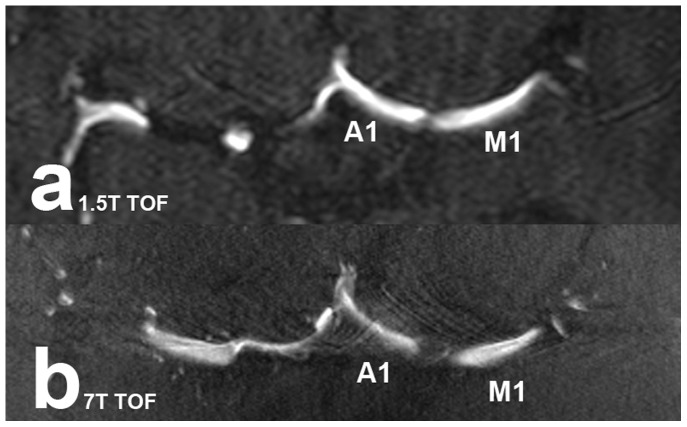
Pulsation artifacts near the carotid bifurcation in TOF MRA scans. The artifacts in 1.5 Tesla (**a**) and 7 Tesla (**b**) TOF MRA scans make vessel depiction more difficult (M1: middle cerebral artery (M1 segment); A1: anterior cerebral artery (A1 segment)).

Wilcoxon matched-pairs two-sided signed-ranks test showed significant differences between 7 Tesla MPRAGE and 7 Tesla TOF MRA (p = 0.0002) ratings and between 7 Tesla MPRAGE and 1.5 Tesla TOF MRA (p = 0.0005) ratings. No significant differences were detected between 7 Tesla TOF MRA and 1.5 Tesla TOF MRA (p = 0.3438) ratings. Figure 3 shows examples of pulsation artifacts for 1.5 Tesla and 7 Tesla TOF MRA sequences.

Vessel-tissue contrast was rated equally good for TOF MRA at both field strengths, with mean values of 4.4 (good) (SE 0.146) for 7 Tesla and 4.2 (good) (SE 0.122) for 1.5 Tesla MRI. MPAGE imaging provided highest vessel-tissue contrast with excellent mean values of 4.9 (SE 0.088). Wilcoxon matched-pairs two-sided signed-ranks test showed significant differences between 7 Tesla MPRAGE and 7 Tesla TOF MRA (p = 0.0063) ratings and between 7 Tesla MPRAGE and 1.5 Tesla TOF MRA (p = 0.0001) ratings. There were no significant differences detected between 7 Tesla TOF MRA and 1.5 Tesla TOF MRA (p = 0.5811) ratings. Quantitative measurements of signal intensities showed significantly higher vessel-tissue contrast for 7 Tesla MPRAGE (p = 0.0018) and 7 Tesla TOF MRA (p = 0.0014) compared to 1.5 Tesla TOF MRA. No difference between vessel-tissue contrast could be detected in 7 Tesla MPRAGE and 7 Tesla TOF MRA (p = 0.5337).

Overall image quality was rated equivalently good (4.3) for 7 Tesla (SE 0.105) and 1.5 Tesla (SE 0.117) TOF MRA and excellent (5.0) (SE 0.025) for 7 Tesla MPRAGE imaging. Wilcoxon matched-pairs two-sided signed-ranks test showed significant differences between 7 Tesla MPRAGE and 7 Tesla TOF MRA (p = 0.0000) ratings and between 7 Tesla MPRAGE and 1.5 Tesla TOF MRA (p = 0.0001) ratings. There were no significant differences detected between 7 Tesla TOF MRA and 1.5 Tesla TOF MRA (p = 0.6250) ratings.

Interobserver accordance was almost perfect (kappa coefficient) for most readings with slightly lower accordance (substantial) for artifact and overall image quality assessment in 1.5 Tesla TOF MRA Details are shown in Table 2.

Table 5 shows mean ratings for artifact delineation, vessel-tissue contrast and overall image quality. Table 6 lists the combined readings of both raters for signal intensities of left middle cerebral artery, adjacent gray matter and calculated vessel-tissue contrast ratio for all subjects.

**Table 5:** Ratings for artifacts. vessel-tissue contrast (VTC) and overall image quality (mean ratings from both readers).

aneurysm	7 Tesla	1.5 Tesla	7 Tesla	7 Tesla	1.5 Tesla	7 Tesla	7 Tesla	1.5 Tesla	7 Tesla
	TOF MRA	TOF MRA	MPRAGE	TOF MRA	TOF MRA	MPRAGE	TOF MRA	TOF MRA	MPRAGE
	artifacts	artifacts	artifacts	VTC	VTC	VTC	image quality	image quality	image quality
1	4.5	5	5	5	3.5	5	4	4.5	4.5
2	4	4.5	5	4.5	3	3.5	4	4	5
3	3.5	5	5	5	4	5	4.5	5	5
4	5	5	5	5	4.5	5	4.5	4.5	5
5	5	5	5	5	4	5	4.5	5	5
6	5	5	5	5	5	5	5	5	5
7	5	3.5	5	5	4	5	4.5	4.5	5
8	5	4	5	5	4	5	5	5	5
9	4	5	5	4.5	5	5	4.5	4.5	5
10	5	5	5	4	5	5	4	4	5
11	5	4.5	5	5	4	5	5	5	5
12	3	4	5	3	5	5	4	4	5
13	3	3.5	5	3	4	4	3	3	5
14	4	5	5	4	4	5	4	4	5
15	4	4	5	4	5	5	4.5	4	5
16	4	4	5	4	4	5	4	4	5
17	4	4	5	4	4	5	4	4	5
18	4	4	5	4	4	5	4	4	5
19	4	4	5	4	4	5	4	4	5
20	4	4	5	4	4	5	4	4	5
**mean**	**4.3**	**4.4**	**5.0**	**4.4**	**4.2**	**4.9**	**4.3**	**4.3**	**5.0**

5 =  excellent. 4 =  good. 3 =  moderate. 2 =  poor. and 1 =  non-diagnostic vessel delineation.

**Table 6:** Combined readings of both raters for signal intensities of left middle cerebral artery, adjacent gray matter and calculated vessel-tissue contrast ratio for all subjects.

Subject	7T MPRAGE left MCA	7T MPRAGE GM	7T MPRAGE VTCR	7T TOF left MCA	7T TOF GM	7T TOF VTCR	1.5T TOF left MCA	1.5T TOF GM	1.5T TOF VTCR
1	1032	263	0.594	908	145	0.725	486	132	0.573
2	1071	282	0.583	1063	192	0.694	581	142	0.607
3	543	135	0.602	881	216	0.606	409	89	0.643
4	897	204	0.629	897	157	0.702	408	106	0.588
5	773	141	0.691	785	185	0.619	438	128	0.548
6	844	179	0.650	864	176	0.662	519	139	0.578
7	1014	205	0.664	951	193	0.663	532	143	0.576
8	913	188	0.658	1088	154	0.752	485	121	0.601
9	851	141	0.716	851	176	0.657	426	94	0.638
10	998	152	0.736	912	198	0.643	508	105	0.657
11	846	174	0.659	865	202	0.621	470	113	0.612
12	909	240	0.582	921	193	0.654	520	133	0.593
13	858	219	0.593	818	175	0.648	534	149	0.564
14	792	165	0.655	944	209	0.637	411	92	0.634
15	831	141	0.710	902	196	0.643	540	127	0.619
16	926	186	0.665	843	184	0.642	494	108	0.641
**mean**			*0.649*			**0.660**			***0.604***

Vessel-tissue contrast ratio 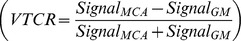 of the middle cerebral artery (MCA) were assessed in correlation to surrounding gray matter (GM) for 7 Tesla (T) magnetization prepared rapid acquisition gradient echo (MPRAGE) sequences, 7T time-of-flight (TOF) sequences and for 1.5T TOF sequences. Therefore, regions of interest (ROI) were defined in the largest diameter of the proximal left M1 segments 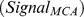 and adjacent gray matter 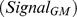. The average diameter for the ROI of the vessel was 3–5 mm; the ROI for brain parenchyma amounted to approximately 1 cm.

standard error  = 0.012, 95% confidence interval [0.623 – 0.675].

standard error =  0.010, 95% confidence interval [0.639 – 0.681].

standard error =  0.008, 95% confidence interval [0.587 – 0.622].

## Discussion

With DSA remaining to be the gold standard, 1.5 Tesla TOF MRA has evolved to become a reliable and equivalent non-invasive technique for detection and follow-up of UIA larger than 3 mm [1], [23]–[27]. The increase in SNR and CNR affiliated to the increase in magnetic field strength has been shown to result in superior vessel (disease) assessment at 3 Tesla compared to 1.5 Tesla [26]. With further increase of the field strength to 7 Tesla, the combination of the associated increase in SNR (up to 4–5 fold higher than at 1.5 Tesla) and longer T1 relaxation times [28] are known to offer an improved vessel-tissue contrast based on more efficient background tissue suppression [29]. Studies in healthy volunteers at 7 Tesla have shown superior vessel delineation compared to 1.5 Tesla [30], [31]. Furthermore, patient studies demonstrated the high diagnostic ability and superiority of 7 Tesla non-enhanced TOF MRA for evaluation of intracranial vasculature and aneurysm detection compared to DSA and/or 1.5 Tesla TOF MRA [13], [32].

Initial studies of 7 Tesla non-enhanced T1w brain imaging revealed an incidental finding, by means of a homogeneously hyperintense signal of arterial vasculature. This incidental finding bears a strong diagnostic potential for non-enhanced high-quality vessel imaging at 7 Tesla, as demonstrated in numerous studies [18], [32]–[36]. In a study presented at the ISMRM 2010, Grinstead et al. analyzed the primary source of the hyperintense vessel signal. Their investigations showed an association of the high vessel signal to the lack of body RF transmit coils at 7 Tesla, resulting in the utilization of head coils for transmit and receive. Hence, non-selective infrared pulses effectively become slab-selective infrared-pulses. Furthermore a combination of steady state and inflow effects seems to be accountable.

To investigate the diagnostic ability of T1w non-enhanced 7 Tesla MRI, Maderwald et al. [32] published an intra-individual comparison trial of 7 Tesla TOF MRA, VIBE imaging (three-dimensional volume interpolated breath hold examination) and MPRAGE imaging of the intracranial vasculature in 25 subjects. Their results demonstrated the superiority of MPRAGE imaging in the assessment of non-enhanced vasculature, providing high-quality delineation of all vessel segments and least impairment due to intraluminal signal variations. Furthermore, MPRAGE and VIBE imaging offered full brain coverage in contrast to TOF MRA. Zwanenburg et al. [37] confirmed the high diagnostic potential of non-enhanced MPRAGE MRI at 7 Tesla, yielding excellent assessment of cerebral perforating arteries and related anatomical parenchymatous structures. In another recent study, the potential diagnostic benefit of the application of contrast agent to 7 Tesla MPRAGE MRI was investigated. The study results revealed only minor non-significant improvement based on the administration of contrast agent, underlining the high-diagnostic potential of non-enhanced 7 Tesla MPRAGE MRI [36].

Based on these previous study results, we decided to include non-enhanced MPRAGE imaging to our 7 Tesla protocol and compare its diagnostic ability to 7 Tesla and 1.5 Tesla TOF MRA regarding the assessment of intracranial aneurysms and their related features. Our study results go in line with previous publications regarding the superiority of 7 Tesla TOF MRA over 1.5 Tesla TOF MRA as well as 7 Tesla MPRAGE over 7 Tesla and 1.5 Tesla TOF MRA. However, while previous studies mainly focused on the evaluation of the overall image quality and overall delineation of the aneurysms, our study results deepen the assessment of the diagnostic ability based on a dedicated analysis of numerous aneurysm features and image quality parameters. Due to its high spatial resolution and excellent vessel-to-tissue contrast MPRAGE MRI offered best delineation of all assessed aneurysm features. It also yielded highest scores in overall image quality and least artifact impairment with significant difference to TOF MRA at 7 Tesla and 1.5 Tesla. Furthermore, aside from excellent vessel delineation, it also offers the potential for simultaneous high quality assessment of related anatomical parenchymatous structures and full brain coverage. While 7 Tesla TOF MRA yielded superior diagnostics of the aneurysm dome and neck over 1.5 Tesla, it was slightly inferior in the assessment of the parent vessel. This inferiority was mainly due to amplified intraluminal signal variations at 7 Tesla, resulting in impaired parent vessel delineation.

Clearly, our study is not free of limitations. The study group comprised a rather small population of 16 patients with a total of 20 untreated IA. Nevertheless, to our knowledge this is one of the largest neurosurgical patients cohorts suffering from UIA scanned at 7 Tesla and 1.5 Tesla MRI, published in literature. Further investigations with larger patient cohorts, also including patients with clipped or coiled intracranial aneurysms should be the focus of future studies. Ultrahighfield imaging in patients with treated aneurysms has been a restricted so far, as neither Guglielmi detachable coils nor aneurysm clips are certified for 7 Tesla MR imaging. Nevertheless, first promising preliminary results on implant safety in cerebral 7 Tesla MRI have been recently demonstrated {Kraff, 2013#1639}{Noureddine, 2012#1640}{Noureddine, 2013#1641}. Future studies on the diagnostic potential of 7 Tesla MRI for follow-up of coiled aneurysms would be of high scientific and clinical interest with special focus on aneurysm recanalization and its precise detection, comparing MRA at different magnetic field strengths to DSA.

Furthermore, another limitation is posed by the lack of a comparison to the diagnostic gold standard, in terms of digital subtraction angiography. However, with MRA offering equivalent non-invasive vessel diagnostics to DSA, particularly applied for aneurysm monitoring (as in our patient cohort), the main focus of this trial was set on a direct comparison of the diagnostic ability of different magnetic field strengths. Finally, as known from previous studies, 3 Tesla MRA is considered to provide improved vessel delineation over 1.5 Tesla. Hence, a comparison of 7 Tesla MRA to 3 Tesla MRA instead of 1.5 Tesla imaging would have been desirable. Unfortunately, this was not applicable due to availability reasons in this clinical setting. Nevertheless, 1.5 Tesla MRI is still considered the worldwide clinical standard. Hence, in order to investigate the diagnostic ability of 7 Tesla MRA, a comparison to the clinical worldwide standard (1.5 Tesla) may be a fair comparison after all.

In conclusion, we believe our study demonstrates the superiority of 7 Tesla MRA over 1.5 Tesla MRA and underlines the high diagnostic potential of 7 Tesla non-enhanced MPRAGE imaging for assessment, screening and follow-up of UIA.
